# Annual and Analytical Cyclopædia of Practical Medicine

**Published:** 1899-08

**Authors:** 


### Bibliography.

Annual and Analytical Cyclopædia of Practical Medicine. By Charles E. de M. Sajous, M.D., and One Hundred Associate Editors. Assisted by Corresponding Editors, Collaborators, and Correspondents. Illustrated with Chromo-Lithographs, Engravings, and Maps. Volume III. The F. A. Davis Company, Publishers. Philadelphia, New York, Chicago, 1899.

In the previous notices of this most valuable cyclopædia the reviewer has given but the one opinion possible, and that has been that each succeeding volume confirms the views heretofore expressed,—that they are the most satisfactory series in practical medicine in the English language with which he is familiar.

The editors and associate editors are all authorities in their special work, and the reader feels that in consulting them he reaches the highest standard of excellence. The editor, in briefly enumerating some of these, says, "The articles on 'Infantile Myxœdema (Cretinism),' by Professor Osler and Dr. Norton, of Baltimore; 'Exophthalmic Goitre,' by Professor Putnam, of Boston; and 'Goitre,' by Professor Adami, of Montreal, thus form a trio which may be said to point to much of the progress that is to attend medicine in the near future."

The subject of gout has of recent years attracted wide attention in dentistry, as it has been held by some to be the producing cause of several of the gingival and peridental inflammations, especially that of pyorrhœa alveolaris. The article upon this disease is from the pen of F. Levison, Copenhagen, and it may be of interest to our readers to quote his opinion as to the treatment. He sums up this as follows: "In all cases the diet is to be regulated with a view to sustain the forces of the patient without allowing any excess of food; the patient is to be advised to limit the use of alcoholic stimulants and to avoid equally excess of work and of enjoyments, whereas bodily exercise and open-air life are useful. The patient ought to drink pure water of some aërated spring in sufficient quantity to keep the daily excretion of urine from three to four pints; if the urine be strongly acid and liable to precipitation of uric acid crystals, the administration of small doses of some alkaline drug or spring should be resorted to to diminish the acidity and render the urine limpid.

"The gouty attacks are treated by rest, somewhat reduced *régime,* anodynes, if necessary, and colchicum; in the free intervals the resin of guaiac will be of use. The stiffness of the gouty joints and the tophi are treated by the dietetic introduction of lithia, by the hot-air bath, and by massage.

"A visit to some spring where the application of hot baths, douches, and massage are combined with the use of some aërated spring and good vivifying air will be of use to restore the forces and the spirits of the patient. Also a sojourn in some dry and hot climate is advisable, as well for the specific gouty symptoms as for the disease of the kidneys, which is the constant companion of gout."

The third volume opens with "Dislocations" and closes with "Infantile Myxœdema."

The type, printing, and general make-up corresponds with previous volumes, and is beyond criticism.

